# Unsupervised segmentation of greenhouse plant images based on modified Latent Dirichlet Allocation

**DOI:** 10.7717/peerj.5036

**Published:** 2018-06-28

**Authors:** Yi Wang, Lihong Xu

**Affiliations:** College of Electronics and Information Engineering, Tongji University, Shanghai, China

**Keywords:** Latent Dirichlet Allocation, Word-document assignment, Mean-shift, Optimal bandwidth search, Plant segmentation

## Abstract

Agricultural greenhouse plant images with complicated scenes are difficult to precisely manually label. The appearance of leaf disease spots and mosses increases the difficulty in plant segmentation. Considering these problems, this paper proposed a statistical image segmentation algorithm MSBS-LDA (Mean-shift Bandwidths Searching Latent Dirichlet Allocation), which can perform unsupervised segmentation of greenhouse plants. The main idea of the algorithm is to take advantage of the language model LDA (Latent Dirichlet Allocation) to deal with image segmentation based on the design of spatial documents. The maximum points of probability density function in image space are mapped as documents and Mean-shift is utilized to fulfill the word-document assignment. The proportion of the first major word in word frequency statistics determines the coordinate space bandwidth, and the spatial LDA segmentation procedure iteratively searches for optimal color space bandwidth in the light of the LUV distances between classes. In view of the fruits in plant segmentation result and the ever-changing illumination condition in greenhouses, an improved leaf segmentation method based on watershed is proposed to further segment the leaves. Experiment results show that the proposed methods can segment greenhouse plants and leaves in an unsupervised way and obtain a high segmentation accuracy together with an effective extraction of the fruit part.

## Introduction

Plant phenotype analysis based on image processing has been a popular application field of agricultural computer vision in recent years. An automatic, high-throughput, accurate and rapid imaging technique and processing method for plant phenotypic analysis will not only monitor the growth of plants, but also lay a visual foundation for the optimization of an intelligent greenhouse environment control system. It is a benefit to gene breeding, environment regulation, cultivation management, and the estimation of yield and quality. In the literature, the convolutional neural network (CNN) has been applied to agriculture, however, a large amount of reliable training data is needed which becomes an urgent problem. For the analysis of greenhouse plants, it is an important process to get enough high-quality labelled images. In this regard, a well-segmented result of the plant and leaves can help in labelling the image quickly and accurately.

There are various imaging methods in the literature, among which a large amount of segmentation methods are developed from different aspects. Currently, there are several common methods to collect information of plants: (1) typical 2D color camera, (2) RGB-D camera, including stereo camera, ToF camera, structured light Kinect and laser scanner, (3) spectrometer, and (4) airborne laser radar. It is known that 2D imaging technologies are simple, low-cost and non-destructive, which can be suitable for plant phenotype analysis such as studying the growth of plant leaves, tracing their position and orientation, classifying new leaves from old ones, examining growth regulation and evaluating environmental stress if an accurate result of leaf segmentation is obtained (Scharr et al., 2016). The 2D imaging technologies lay a visual foundation for the analysis and warning of leaf disease as well.

For plant segmentation, the use of color in combination with depth images or multi-view images for supervised or unsupervised segmentation is a popular practice (Alenya, Dellen & Torras, 2011; Song et al., 2007; Teng, Kuo & Chen, 2011). Additionally, researchers have proposed some methods of segmenting plants from their background, in order to handle leaves with lesions (Zou et al., 2015; Valliammal & Geethalakshmi, 2012) and those without lesions (Zhang, Zhang & Guo-Hong, 2017; Wang, Wang & Cui, 2011). The background of the plants is artificial or relatively brief. One method for the detection of leaf disease spots is to directly extract the lesion part from the leaf of interest (Ma et al., 2017), while the other method is to separate a whole leaf from the complex background and extract the lesion part subsequently (Fang, Lu & Lisi, 2014). The latter can provide a better view to observe the site of lesions and analyze the severity of the disease. For single leaf segmentation, methods can be roughly categorized into four classes: shape model constraints (Manh et al., 2001; Mezzo et al., 2007; Cerutti et al., 2011; Sogaard, 2005; Persson & Astrand, 2008), boundary information detection (Valliammal & Geethalakshmi, 2011; Noble & Brown, 2008; Tang et al., 2009; Lee & Slaughter, 2004), depth information integration (Guo & Xu, 2017; Alenya et al., 2013; Xia et al., 2015; Sanz-Cortiella et al., 2011) and machine learning methods (Zheng, Zhang & Wang, 2009; Meunkaewjinda et al., 2008; Hernández-Rabadán, Guerrero & Ramos-Quintana, 2012; Pape & Klukas, 2015). Recently, the end-to-end recurrent neural network (RCNN) architecture (Ren & Zemel, 2016; Romera-Paredes & Torr, 2016), using an attention mechanism to model a human-like counting process, has provided a new way to handle occlusion by proceeding sequentially. Some of the methods above are mainly utilized in the classification of plants by extracting one complete leaf from a plant, while other methods segment all leaves in the image. Compared with the former, the latter methods have advantages in applications like genome-environment-phenotype synergistic analysis which cannot only rely on the information of one single leaf.

In recent years, more and more statistical methods have been applied in the image processing field. The Latent Dirichlet Allocation (LDA) model (Blei, Ng & Jordan, 2003) introduces a parameter *θ* which obeys Dirichlet distribution on the basis of Probabilistic Latent Semantic Analysis (pLSA) to establish the probability distribution of latent topic variable z. Li & Perona (2005) first applied the LDA model to image segmentation and many improved models came into view later. For example, a Spatial Latent Dirichlet Allocation (SLDA) topic model (Wang & Grimson, 2008) encodes the spatial structure of visual words as a random hidden variable in a better way with LDA’s generative procedure. A spatially coherent latent topic model (Spatial-LTM) (Cao & Li, 2011) provides a unified representation for a spatially coherent bag of word topic models, which can simultaneously segment and classify objects in the case of occlusion and multiple instances. Russell et al. (2006) partition a set of segmented objects into visual object classes using LDA and the visual object classes are further used to assess the accuracy of a segmentation. Reddy, Singhal & Krishna (2014) propose an algorithm that jointly infers the semantic class and motion labels of an object, integrating the semantic, geometric and optical flow based constraints into a Dense-CRF model.

In this paper, we first analyze the difficulties of applying a natural language processing model to image segmentation, and improve the spatial structure encoding of LDA through the word-document assignment strategy. Considering the problems of leaf disease spots and mosses, a spatial LDA image segmentation algorithm based on Mean-shift document bandwidths searching (MSBS-LDA) is proposed and applied to plant segmentation. In view of the problems with tomato fruits and the complicated illumination environment, an improved leaf segmentation method based on watershed is proposed to further segment the leaves. Experimental results show that the proposed methods can achieve a high accuracy of image segmentation.

## Latent Dirichlet Allocation (LDA)

The Latent Dirichlet Allocation (LDA) was first proposed by Blei, Ng & Jordan (2003). It is a topic generation model which contains a three-level structure of word, topic and document (Griffiths & Steyvers, 2004; David, 2010). Both the topics of a document and the words of a topic obey polynomial distribution. It uses the Bag of Words (BoW) model, where each document is regarded as a word frequency vector, such that textual information is transformed into easy-to-model digital information. Therefore, LDA can be used to identify latent topic information in a large document collection or corpus. In recent years, LDA has been widely used in the field of machine vision as an unsupervised machine learning technology, such as target discovery, scene classification, behavior detection and visual surveillance (Wang & Grimson, 2008). However, spatial structures among visual words, which are vital in computer vision issues, are ignored in the language model since BoW forms a huge gap between low-level visual words and high-level semantics.

**Figure 1:**
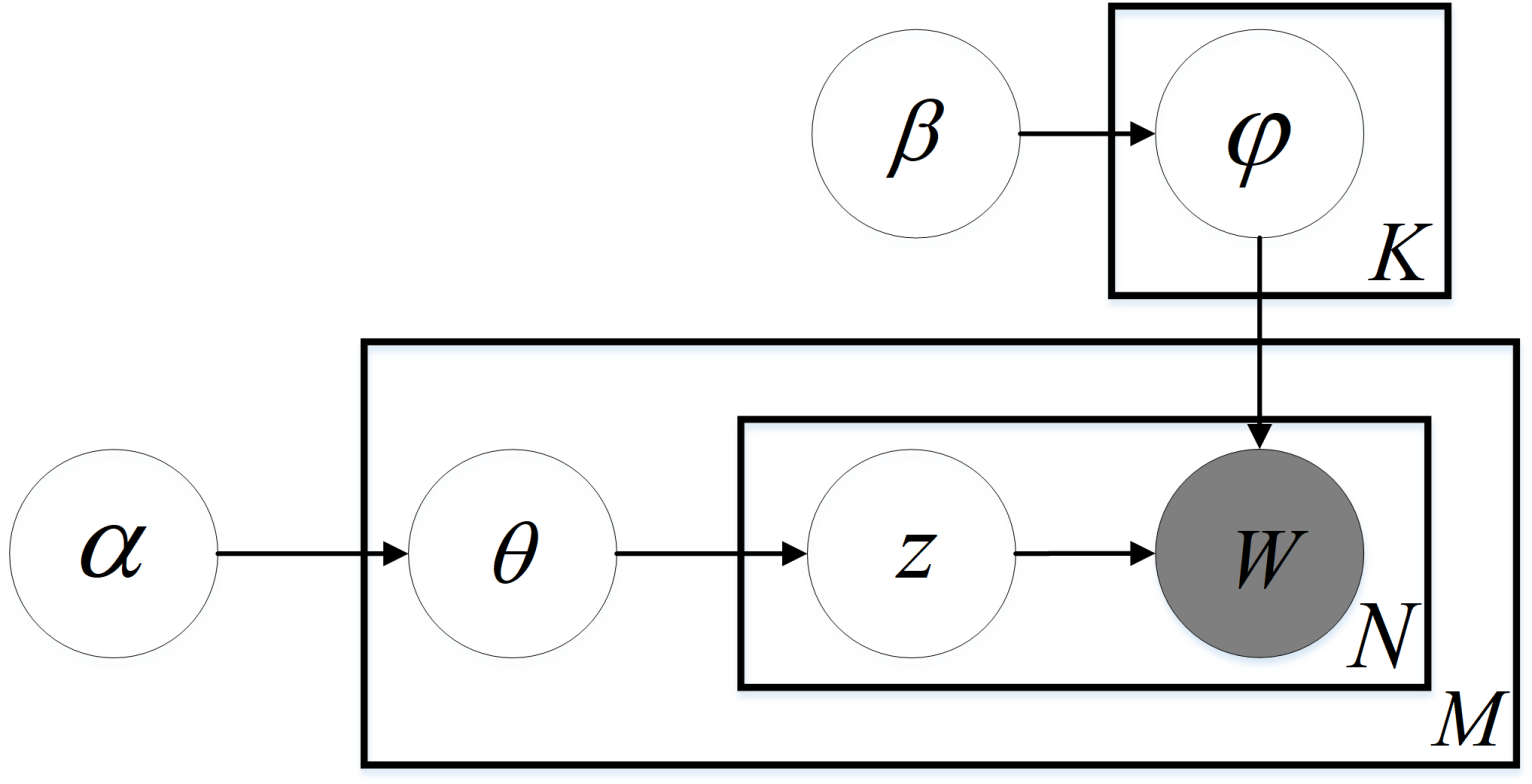
LDA Bayesian network structure.

LDA gives the topic of each document in a document collection or corpus as a probability distribution. It is an unsupervised learning algorithm, which does not need manual annotation of the training set. Only a corpus and the number of topics are demanded. Note that a document can contain more than one topic, and each word in the document is generated by one of the topics. As depicted in the LDA Bayesian network structure (Fig. 1), the generative procedure of a document in LDA can be described as follows:

(1) For a document *i*, its topic distribution *θ*_*i*_ is sampled from Dirichlet priori $Dir \left( \alpha \right) $;

(2) For a word *j* in document *i*, its topic label *z*_*i*,*j*_ is sampled from topic multinomial distribution *θ*_*i*_;

(3) For a topic *z*_*i*,*j*_, its word distribution *ϕ*_*z*_*i*,*j*__ is sampled from Dirichlet priori $Dir \left( \beta \right) $;

(4) The value *w*_*i*,*j*_ of word *j* in document *i* is sampled from word multinomial distribution *ϕ*_*z*_*i*,*j*__;

Thus, the joint distribution of all visible variables and latent variables in the model is:

(1) \begin{eqnarray*}p \left( {w}_{i},{z}_{i},{\theta }_{i},\Phi \left\vert \alpha ,\beta \right. \right) =\prod _{j=1}^{N}p \left( {\theta }_{i} \left\vert \alpha \right. \right) p \left( {z}_{i,j} \left\vert {\theta }_{i} \right. \right) p \left( \Phi \left\vert \beta \right. \right) p \left( {\omega }_{i,j} \left\vert {\varphi }_{{z}_{i,j}} \right. \right) .\end{eqnarray*}

The maximum likelihood estimation for a document’s word distribution can be obtained by integrating *θ*_*i*_, Φ and summing *z*_*i*_, as shown by:

(2) \begin{eqnarray*}p \left( {w}_{i} \left\vert \alpha ,\beta \right. \right) =\int \nolimits _{{\theta }_{i}}\int \nolimits _{\Phi }\sum _{{z}_{i}}p \left( {w}_{i},{z}_{i},{\theta }_{i},\Phi \left\vert \alpha ,\beta \right. \right) .\end{eqnarray*}

According to the maximum likelihood estimation of $p \left( {w}_{i} \left\vert \alpha ,\beta \right. \right) $, the parameters in the model can finally be estimated by Gibbs sampling.

## Spatial LDA

When applied to image segmentation, the modified LDA needs to meet the following two requirements: (1) it should have a good description of local features and (2) it should be equipped with a good spatial summarization ability. The former concentrates on the construction of visual words while the latter focuses on the design of visual documents, making up for the defect of the BoW model. The two processes of local feature description and global structure summarization need to complement each other, leading to the improvement of spatial LDA algorithm.

### Visual words

When we compute visual words, it should be noted that the detailed information of image feature is not the more the better. Excessively high dimension makes it difficult to achieve convergence when learning a dictionary in an unsupervised way, thus hindering LDA to extract latent topics. In this regard, moderate detailed features can be considered as the key to the ability of spatial LDA to describe local information.

Visual words are local descriptors that make up the whole content of an image. They mostly focus on local details instead of global structure or relationships among objects. In order to obtain the local descriptors, images are convolved with a filter bank which consists of three Gaussians, four Laplacian of Gaussians (LoG), and four first order derivatives of Gaussians. According to Winn, Criminisi & Minka (2005), the filter bank has shown to achieve good performance for feature description and object categorization. The three Gaussian kernels (with *σ* = 1, 2, 4) are applied to each CIE L,a,b channel, thus producing nine filter responses. The four LoGs (with *σ* = 1, 2, 4, 8) are applied to the L channel only, thus producing four filter responses. The four derivatives of Gaussians are divided into the two *x*- and *y*-aligned sets, each with two different values of *σ* (*σ* = 2, 4). Moreover, these four derivatives of Gaussians are applied to the L channel only, thus producing four final filter responses. Totally, each pixel in the image has associated a 17-dimensional feature vector.

Afterwards, the image is divided into local patches on a grid and we densely sample a local descriptor for each patch. For the purpose of clustering all the local descriptors, K-means is employed to obtain N cluster centers $ \left( {c}_{1},{c}_{2},\ldots ,{c}_{N} \right) $ which form the visual dictionary $\mathrm{V }= \left\{ {c}_{1},{c}_{2},\ldots ,{c}_{N} \right\} $. The local descriptors *G*_1_, *G*_2_, …, *G*_*M*_ are quantified into visual words according to the dictionary, with the minimum of Euclidean distance between each descriptor and each cluster center, as shown in Eq. (3).

(3) \begin{eqnarray*}{w}_{j}=argmi{n}_{{c}_{i}}{ \left\| {G}_{j}-{c}_{i} \right\| }^{2},i\in \left[ 1,N \right] ,j\in \left[ 1,M \right] .\end{eqnarray*}

Thus the original image is converted into data consisting of visual words. In the next step, the visual words (image patches) will be assigned to visual documents (image regions) and further clustered into different classes (semantic objects).

### Visual documents

After establishing the visual BoW, we need to further design the visual documents. The traditional LDA assumes that the corpus is a BoW model, which neglects objects and their spatial relationship. When we determine which visual document a pixel is assigned to, it actually contains some design of spatial information about the image. Therefore, we call the modified LDA with visual word-document assignment, spatial LDA. On the other hand, the design of visual documents, such as structure and size, generally imply specific visual assumptions on the image. Whether these assumptions are strong or not, it is directly related to the quality of image segmentation. For example, if we take the whole image as a document, it is assumed that if two patches belong to the same object, they often appear in the same image (Assumption 1). This assumption is reasonable but not rigorous.

In these regards, the design of visual documents should comply with the following two principles. (1) First, visual documents are required to depict spatial information reasonably and focus on certain objects in the image space. A document expressing many different objects leads to the loss of important edge information, since image patches in a document tend to have the same topic label, while a document with few words does not have enough words to describe a certain topic. (2) Second, it is supposed that visual documents have a certain tolerance to feature differences in an object, in order to deal with a situation whose features vary widely within an object, like disease spots on leaves. The co-occurrence information of LDA, to some extent, has been able to classify different components, belonging to one object but with feature differences, into a topic. But it requires a large number of complete observation samples, which tends to make the algorithm time-consuming and accounting for storage, thus reducing the efficiency of spatial LDA. To alleviate this problem, we further enhance this co-occurrence information in the word-document assignment by blurring different parts of an object during the design of visual words, which can eliminate unnecessary weak edges and preserve critical strong edges.

Before discussing the unsupervised learning method Mean-shift Bandwidths Searching Latent Dirichlet Allocation (MSBS-LDA), we present two kinds of word-document assignment strategies that have some limitations on the above requirements.

#### Rectangular documents

If the document of a patch only includes other patches falling within its neighborhood, instead of the whole image, it corresponds to a better assumption: if two types of image patches are from the same object class, they are not only often in the same image but also close in space (Assumption 2). Hence, considering the tolerance to feature differences, two kinds of grid-based spatial information encoding methods are proposed.

Firstly, a rectangular region, which is taken as the document, is defined based on the grid with fixed size of *R*_1_ × *R*_2_. The local patches in each region are quantized into visual words according to the dictionary, and subsequently, assigned to their documents. In the case that some patches are very close in space but assigned to different documents, an encoding scheme with overlapping rectangular regions is considered here. On the contrary, there is no overlapping region for another encoding scheme. That is the main difference between the proposed two encoding methods in this section. The detailed steps of image segmentation are as follows:

(1) The image is divided into local patches on a grid. Then local descriptors are calculated for each patch, and subsequently quantified into visual words {**w**} according to the vocabulary $\mathrm{V }= \left\{ {c}_{1},{c}_{2},\ldots ,{c}_{N} \right\} $;

(2) The image is divided into rectangular regions of *R*_1_ × *R*_2_ size. Every two adjacent rectangular regions do not overlap (or overlap $ \left( {R}_{1}\times {R}_{2} \right) /2$), generating a number of visual documents {**d**};

(3) According to the visual word frequency histogram *H*^*d*^, d = 1, …, D, a corpus C is established for LDA;

(4) Suppose that the number of segments is K, a LDA model with K topics is trained based on the corpus C to obtain the probability that pixels of each region belong to K topics, namely ${\varphi }_{i}^{d}=\mathrm{P} \left( z{|}{w}_{i},d \right) $. Note that, for the words of overlapping documents, the probability *φ*_*i*_ is the average of ${\varphi }_{i}^{d}$ obtained from all the corresponding regions, as shown in Eq. (4):

(4) \begin{eqnarray*}{\varphi }_{i}=\exp \nolimits \left( \mathrm{mean} \left( \sum _{d,i\in d}\log \nolimits {\varphi }_{i}^{d} \right) \right) \end{eqnarray*}

(5) Each pixel is classified to a topic with the largest probability value of *φ*_*i*_, thus all pixels of the image are partitioned into K classes.

With the two different encoding scheme of rectangular documents described in this section, two versions of spatial LDA segmentation algorithms are proposed, namely Non-overlapping Rectangular Documents index LDA (NR-LDA for short) and Overlapping Rectangular Documents index LDA (OR-LDA for short).

#### Super-pixel documents

Based on the space proximity assumption (i.e., Assumption 2), we attempt to design visual documents regarding the characteristics of the image itself. Since super-pixels combine pixels into meaningful atomic regions, they can be used to replace the rigid structure of the grid-based rectangular regions. Generally, the over-segmented super-pixel regions can retain most of the valid information for further image segmentation, and do not destroy the edge information of objects. Thus we further consider a super-pixel based spatial information encoding method in our research.

First, the basic information of the image is abstracted by super-pixels, thereby clustering a pixel-level image to a district-level map. These irregular pixel blocks, which are composed of adjacent pixels with similar texture, color, brightness and so on, are regarded as a corpus C. Accordingly, the corpus C contains a series of regions, each of which includes visual words corresponding to the pixels, forming a tree structure of spatial LDA. The subsequent image segmentation steps are described in steps (4) and (5) of section ‘Rectangular documents’ without the detail of the overlapping case. Here we use SLIC (Simple Linear Iterative Clustering) to implement the super-pixel encoding. Compared with other super-pixel segmentation methods, SLIC is superior in operating speed, super-pixel compactness and contour retention (Achanta et al., 2012).

For simplicity, the LDA segmentation algorithms based on super-pixel documents is named as Simple Linear Iterative Clustering Documents index LDA (SLIC-LDA for short).

#### Limitation analysis

By using rectangular regions and super-pixel regions, the word-document assignments usually have the following main limitations:

(1) In case of the rectangular documents, some parameters should be fixed such that they take the spatial structure of the image as a fixed and explicit variable. Although the documents of overlapping regions have some tolerance to the feature differences of an object, the rigid structure based on grid is separated from the characteristics of the image. Every document contains the same number of visual words and the size of all the documents is uniquely determined by the rectangular area. This kind of word-document assignment cannot describe the spatial information of objects in the image naturally.

(2) In the case of the super-pixel documents, they usually measure a cluster using the traditional distance, which is, however, unreasonable in some cases. Also, they replace the rigid structure of grid-based rectangular regions with some meaningful atomic regions, and accordingly, some unnecessary weak edge information of the object in the image may be introduced in addition to most valid edge information that has been retained. Besides, the over-segmentation result actually weakens the documents’ tolerance to feature differences, which ought to make spatial LDA reduce the co-occurrence information of the same object in the word-document assignment.

Moreover, the design of rectangular documents and super-pixel documents can only classify visual words located within a certain distance into one document. If the object occupies more pixels and the distance between pixels is farther, it reflects the inaccuracy of Assumption 2. In view of the problems above, we propose a spatial LDA segmentation algorithm based on Mean-shift documents in the next section and compare traditional LDA to spatial LDAs with different designs of documents then.

## MSBS-LDA

For the SLDA algorithm (Wang & Grimson, 2008), each document is represented by a point in the image, assuming that its region covers the whole image. If an image patch is close to a document, it has a high probability to be assigned to that document. To describe the word-document mechanism, several parameters are introduced as follows:

(1) *d*_*j*_: a hidden variable indicates which document word *j* is assigned to;

(2) ${c}_{i}^{d}$: a hyper-parameter for document *i* known as a priori;

(3) ${g}_{i}^{d}$: the index of the image where document *i* is placed;

(4) $ \left( {x}_{i}^{d},{y}_{i}^{d} \right) $: the location of document *j*;

(5) ${c}_{j}= \left( {g}_{j},{x}_{j},{y}_{j} \right) $: storing location $ \left( {x}_{j},{y}_{j} \right) $ and image index *g*_*j*_ of word *j*;

In the generation procedure of SLDA, for a word *j*, a random variable *d*_*j*_ is sampled from prior $\mathrm{p} \left( {d}_{j}{|}\eta \right) $ indicating which document word *j* is assigned to and a uniform prior is used. The image index and location of word *j* is sampled from distribution $p \left( {c}_{j}{|}{c}_{{d}_{j}}^{d},\sigma \right) $, and a Gaussian kernel is chosen:

(5) \begin{eqnarray*}p \left( \left( {g}_{j},{x}_{j},{y}_{j} \right) \left\vert \left( {g}_{{d}_{j}}^{d},{x}_{{d}_{j}}^{d},{y}_{{d}_{j}}^{d} \right) \right. ,\sigma \right) \propto {\delta }_{{g}_{{d}_{j}}^{d}} \left( {g}_{j} \right) \exp \nolimits \left\{ - \frac{{ \left( {x}_{{d}_{j}}^{d}-{x}_{j} \right) }^{2}+{ \left( {y}_{{d}_{j}}^{d}-{y}_{j} \right) }^{2}}{{\sigma }^{2}} \right\} \end{eqnarray*}

where $p \left( {c}_{j}{|}{c}_{{d}_{j}}^{d},\sigma \right) =0$ if the word and the document are not in the same image. In the procedure of parameter estimation, *z*_*j*_ and *d*_*j*_ are sampled through a Gibbs sampling procedure integrating out *ϕ*_*k*_ and *θ*_*i*_. The conditional distribution of *z*_*j*_ given *d*_*j*_ is the same as in LDA, which is given by:

(6) \begin{eqnarray*}p \left( {z}_{j}=k{|}{d}_{j}=i,{d}_{-j},{z}_{-j},w,\alpha ,\beta \right) \propto \frac{{n}_{-j,{w}_{j}}^{ \left( k \right) }+{\beta }_{{w}_{j}}}{\sum _{w=1}^{W} \left( {n}_{-j,w}^{ \left( k \right) }+{\beta }_{w} \right) } \cdot \frac{{n}_{-j,k}^{ \left( i \right) }+{\alpha }_{k}}{\sum _{{k}^{{^{\prime}}}=1}^{K} \left( {n}_{-j,{k}^{{^{\prime}}}}^{ \left( i \right) }+{\alpha }_{{k}^{{^{\prime}}}} \right) } \end{eqnarray*}

The method attempts to borrow the language model to image segmentation, for which a uniform prior is applied to determine which document a word is assigned to and a Gaussian kernel is adopted to describe the location of the word. Inspired by this idea, we consider the local maximum points of probability density function (P.D.F) of an image as documents, and the density estimation should be estimated with a nonparametric method due to the fact that the distribution of image data has no fixed pattern.

As a feature space analysis method, Mean-shift (Comaniciu & Meer, 2002; Comaniciu, Ramesh & Meer, 2000) attempts to find the local maximum points of P.D.F in a joint space, and applies nonparametric kernel density estimation (KDE) with smooth effect to density estimation, which provides a new way for word-document assignment of LDA. Thus we propose a modified LDA segmentation algorithm, namely Mean-shift Bandwidths Searching Latent Dirichlet Allocation (MSBS-LDA), for which Mean-shift is adopted to determine which document a word is assigned to. Moreover, the documents are represented by the modes of P.D.F and instead of applying Gibbs sampling to parameter estimation, it applies kernel density estimation to encode image information.

The pixels of each image include two types of information in coordinate space (*x*^*s*^, $ \left[ px,py \right] $) and color space (*x*^*r*^, $ \left[ l,u,v \right] $) such that the five-dimensional joint feature space $ \left[ px,py,l,u,v \right] $ is constituted. For a point *x*, Mean-shift iteratively searches its mode *y* in the joint space and assigns the color value of the mode to itself, that is *x*^*r*^ = *y*^*r*^. If we set the footprint of *x*_*i*_ to $ \left\{ {y}_{i,0},{y}_{i,1},{y}_{i,2},\ldots ,{y}_{i,k},\ldots ,{y}_{i,c} \right\} $ then *y*_*i*,0_ = *x*_*i*_ at the beginning and it converges to the mode *y*_*i*,*c*_. The procedure of mode detection is as follow:

(1) Screen points close to ${y}_{i,k}^{s}$ in the coordinate space to the next step. Note that *h*_*s*_ is the kernel function bandwidth in coordinate space.

(2) Use the points survived to calculate the center of gravity and move towards it according to Eq. (7).

(7) \begin{eqnarray*}{y}_{i,k+1}^{s}= \frac{\sum _{n=1}^{N}{x}_{n}^{s}g \left( { \left\| \frac{{x}_{n}^{r}-{y}_{i,k}^{r}}{{h}_{r}} \right\| }^{2} \right) }{\sum _{n=1}^{N}g \left( { \left\| \frac{{x}_{n}^{r}-{y}_{i,k}^{r}}{{h}_{r}} \right\| }^{2} \right) } \end{eqnarray*}

where *h*_*r*_ is the kernel function bandwidth in color space.

(3) Decide if the stopping condition is met or if the number of iterations exceed the maximum limit. If so, stop the search and turn to next step, otherwise, return to step (1) and start from *y*_*k*+1_. The stopping condition for search is determined by:

(8) \begin{eqnarray*} \left\{ \begin{array}{@{}l@{}} \displaystyle {y}_{i,k+1}^{s}={y}_{i,k}^{s}\\ \displaystyle \left\| {y}_{i,k+1}^{r}-{y}_{i,k}^{r} \right\| \leq thr \end{array} \right. \end{eqnarray*}

(4) Assign the color ${y}_{i,c}^{r}$ of the mode *y*_*i*,*c*_ to the starting point *x*_*i*_∕*y*_*i*,0_, namely ${x}_{i}^{r}\leftarrow {y}_{i,c}^{r}$.

For density estimation at point *x*, the non-parametric density estimation is computed as:

(9) \begin{eqnarray*}\hat {f} \left( x \right) = \frac{1}{N{h}^{d}} \sum _{n=1}^{N}K \left( \frac{{x}_{n}-x}{h} \right) = \frac{1}{N{h}^{d}} \sum _{n=1}^{N}{c}_{k,d}k \left( { \left\| \frac{{x}_{n}-x}{h} \right\| }^{2} \right) \end{eqnarray*}

where $\mathrm{K} \left( x \right) $ is the kernel function, for which the radially symmetric function is used here such that $\mathrm{K} \left( x \right) ={c}_{k,d}k \left( { \left\| x \right\| }^{2} \right) $. Note that, *c*_*k*,*d*_ is a normalization constant, making $\int _{{R}^{d}}K \left( x \right) dx=1$, and $\mathrm{k} \left( x \right) $ is the profile for $\mathrm{K} \left( x \right) $. The Epanechnikov Kernel is adopted and its profile is given by:

(10) \begin{eqnarray*}\begin{array}{@{}l@{}} \displaystyle k \left( x \right) = \left\{ \begin{array}{@{}ll@{}} \displaystyle 1-x&\displaystyle 0\leq x\leq 1\\ \displaystyle 0&\displaystyle \text{otherwise} \end{array} \right. \\ \displaystyle {K}_{E} \left( x \right) = \left\{ \begin{array}{@{}ll@{}} \displaystyle \frac{1}{2} \left( d+2 \right) \left( 1-{ \left\| x \right\| }^{2} \right) &\displaystyle \left\| x \right\| \leq 1\\ \displaystyle 0&\displaystyle \text{otherwise} \end{array} \right. \end{array}\end{eqnarray*}

In addition, the density gradient estimation for point *x* is computed as:

(11) \begin{eqnarray*}\hat {\nabla }{f}_{h,K} \left( x \right) = \frac{2{c}_{k,d}}{N{h}^{(d+2)}} \sum _{n=1}^{N} \left( {x}_{n}-x \right) \left[ -{k}^{{^{\prime}}} \left( { \left\| \frac{ \left( {x}_{n}-x \right) }{h} \right\| }^{2} \right) \right] \end{eqnarray*}

We know where to move and then the step length is determined by:

(12) \begin{eqnarray*}\begin{array}{@{}lll@{}} \displaystyle \hat {\nabla }{f}_{h,K} \left( x \right) &\displaystyle =&\displaystyle \frac{2{c}_{k,d}}{N{h}^{(d+2)}} \sum _{n=1}^{N} \left( {x}_{n}-x \right) \left[ -{k}^{{^{\prime}}} \left( { \left\| \frac{ \left( {x}_{n}-x \right) }{h} \right\| }^{2} \right) \right] \\ \displaystyle &\displaystyle =&\displaystyle \frac{2{c}_{k,d}}{N{h}^{(d+2)}} \sum _{n=1}^{N} \left( {x}_{n}-x \right) g \left( { \left\| \frac{ \left( {x}_{n}-x \right) }{h} \right\| }^{2} \right) \\ \displaystyle &\displaystyle =&\displaystyle \frac{2{c}_{k,d}}{N{h}^{(d+2)}} \left[ {x}_{n}\sum _{n=1}^{N}g \left( { \left\| \frac{ \left( {x}_{n}-x \right) }{h} \right\| }^{2} \right) -x\sum _{n=1}^{N}g \left( { \left\| \frac{ \left( {x}_{n}-x \right) }{h} \right\| }^{2} \right) \right] \\ \displaystyle &\displaystyle =&\displaystyle \frac{2{c}_{k,d}}{{h}^{2}{c}_{g,d}} _{C} \left[ \frac{{c}_{g,d}}{N{h}^{d}} \sum _{n=1}^{N}g \left( { \left\| \frac{ \left( {x}_{n}-x \right) }{h} \right\| }^{2} \right) \right] _{{\hat {f}}_{h,G} \left( x \right) } \left[ \frac{\sum _{n=1}^{N}{x}_{n}g \left( { \left\| \frac{ \left( {x}_{n}-x \right) }{h} \right\| }^{2} \right) }{\sum _{n=1}^{N}g \left( { \left\| \frac{ \left( {x}_{n}-x \right) }{h} \right\| }^{2} \right) } -x \right] _{{m}_{h,G} \left( x \right) } \end{array}\end{eqnarray*}

where ${\mathrm{m}}_{h,G} \left( x \right) $ is the mean shift vector. It is a directional vector, which corresponds to the gradient direction. For a point *x* moving to *x’* in the gradient direction, the new coordinate is:

(13) \begin{eqnarray*}{x}^{{^{\prime}}}=x+{m}_{h,G}= \frac{\sum _{n=1}^{N}{x}_{n}g \left( { \left\| \frac{{x}_{n}-x}{h} \right\| }^{2} \right) }{\sum _{n=1}^{N}g \left( { \left\| \frac{{x}_{n}-x}{h} \right\| }^{2} \right) } .\end{eqnarray*}

As we can see from the above steps, the document assignment of word *i* is hugely affected by the bandwidths *h*_*s*_ and *h*_*r*_ of multivariate kernel density estimation. When we fix *h*_*s*_ and increase *h*_*r*_ monotonically within a certain range, the number of clusters decreases. A large *h*_*r*_ leads to inadequate documents (regions) while a small *h*_*r*_ results in excessive documents (regions). Similarly, the number of clusters decreases when *h*_*r*_ is fixed and *h*_*s*_ increases. In this regard, we propose to use the word frequency statistics and structural features of the image to find the required parameters $ \left( {h}_{s},{h}_{r} \right) $. According to the meaning of the coordinate scale parameter *h*_*s*_, we count the word frequency *w*_*fren*_ of the quantified visual words in accordance with the LDA dictionary. Then *h*_*s*_ is calculated on the basis of the proportion of the first major word by Eq. (14) where C is a constant in [1.41, 1.67]. For the color scale parameter *h*_*r*_, we iteratively call the LDA segmentation procedure based on Mean-shift documents to obtain an optimal result. The mean value of LUV distance between the K classes is calculated by Eq. (15) and the last three $\Delta \bar {E}$ serve as a cut-off condition for the iteration of LDA.

(14) \begin{eqnarray*}& {h}_{s}=1{0}^{C}\times \frac{{w}_{fren}^{ \left( 1 \right) }}{N} \end{eqnarray*}

(15) \begin{eqnarray*}& \Delta \bar {E}= \frac{2\sum _{i=1}^{K-1}\sum _{j=i+1}^{K}\sqrt{{ \left( {\bar {L}}_{i}-{\bar {L}}_{j} \right) }^{2}+{ \left( {\bar {U}}_{i}-{\bar {U}}_{j} \right) }^{2}+{ \left( {\bar {V}}_{i}-{\bar {V}}_{j} \right) }^{2}}}{K \left( K-1 \right) } .\end{eqnarray*}

Under the framework of LDA, the number of topics K is determined manually. After setting *h*_*s*_, we initialize *h*_*r*_ and decrease it with a step of one. In the beginning, Mean-shift obtains few clusters where the structure information is not obvious and changes a lot. Hence, LDA based on these documents leads to mis-segmentation, and the resulting $\Delta \bar {E}$ between the K classes is unstable. During the implementation process, *h*_*r*_ gradually decreases and the number of clusters increases, and under-segments change to over-segments as a result. Thereafter, the mis-segmentation situation of LDA is significantly improved, and $\Delta \bar {E}$ tends to stabilize after a big jump. If *h*_*r*_ is quite small, Mean-shift turns up excessive segments and the structural information is destroyed. Therefore, we aim to find a set of over-segmented Mean-shift documents, which should maintain certain structural description. The qualitative estimate of the value of *h*_*r*_ should fall in a middle range.

To obtain the final segmentation result, we need to consider the stopping criterion for the iteration of LDA. Note that, when under-segments change to over-segments, the Mean-shift documents have a great impact on LDA segmentation result. When *h*_*r*_ falls in a suitable middle range, the LDA segmentation result tends to be stable, and if *h*_*r*_ continues to decrease, the segmentation result is adversely effected. Therefore, we need to find the segmentation result immediately when the stable state is reached. We take it as the condition of convergence that the difference of three consecutive $\Delta \bar {E}$ is less than one. But sometimes there may occur special cases where two consecutive $\Delta \bar {E}$ are close and then spread out or the optimal range of the LDA segmentation result is limited. At this time, we take the segmentation corresponding to the first large jump of $\Delta \bar {E}$ as the optimal result to output. In addition, when *h*_*r*_ decreases within 10, the number of Mean-shift clusters increases rapidly, which leads to an “overleaping” on the number of spatial documents, thus affecting the judgement of convergence. Therefore, when *h*_*r*_ is less than 10, we change the step length to 0.5 to reduce the adverse effect of sudden changes in the document number on the segmentation results.

Based on the calculation above, the topic probability distribution for each pixel can be obtained by LDA given the word-document assignment. The generation process is as follows:

(1) For a document *i*, multinomial parameter *θ*_*i*_ for *K* topics is sampled from Dirichlet priori, ${\theta }_{i}\sim Dir \left( \alpha \right) $;

(2) For a topic *k*, multinomial parameter *ϕ*_*k*_ is sampled from Dirichlet priori, ${\phi }_{k}\sim Dir \left( \beta \right) $;

(3) For a word *j*, its document *d*_*j*_ is determined by the search of kernel function bandwidth $h= \left( {h}_{s},{h}_{r} \right) $ of Mean-shift;

(4) For a word *j*, its topic label *z*_*j*_ is sampled from discrete distribution of document *d*_*j*_, ${z}_{j}\sim Discrete \left( {\theta }_{{d}_{j}} \right) $;

(5) The value *w*_*j*_ of word *j* is sampled from discrete distribution, ${w}_{j}\sim Discrete \left( {\phi }_{{z}_{j}} \right) $.

Based on the analysis above, MSBS-LDA makes an improvement to Assumption 2 and proposes Assumption 3: If two image patches are from the same object class, they often appear in one image, and they belong to the pre-classified document in image space. That is to say, word *i* is likely to be assigned to document *j* if satisfying the following conditions: (1) they are in the same image, (2) word *i* belongs to the same document as its mode, and (3) the mode is found by Mean-shift combined with word frequency statistics and LDA iterative search. The assumption it adds is that a word tends to have the same topic label as other words in the pre-classified document of image space.

To overcome the limitations of two word-document assignments proposed in Sect. ‘Visual documents’, MSBS-LDA makes some breakthroughs in the two major difficulties of document design: image data modelling and document tolerance.

(1) Spatial information of the image is modelled by nonparametric estimation and the filtering effect of Mean-shift enhances the tolerance of the documents to feature differences, leading to a good global summary ability of the algorithm.

(2) Consider that the bandwidth parameters need to be manually adjusted for spatial documents, which is a non-trivial issue in case of real-world problems without domain knowledge. We propose to use word frequency statistics to determine the coordinate space bandwidth parameter *h*_*s*_ and iteratively search the optimal color space bandwidth parameter *h*_*r*_ in combination with the LDA segmentation algorithm of Mean-shift documents. The optimization of word-document assignment can reduce the time and resource consumption and greatly improve the accuracy of LDA segmentation result.

**Figure 2:**
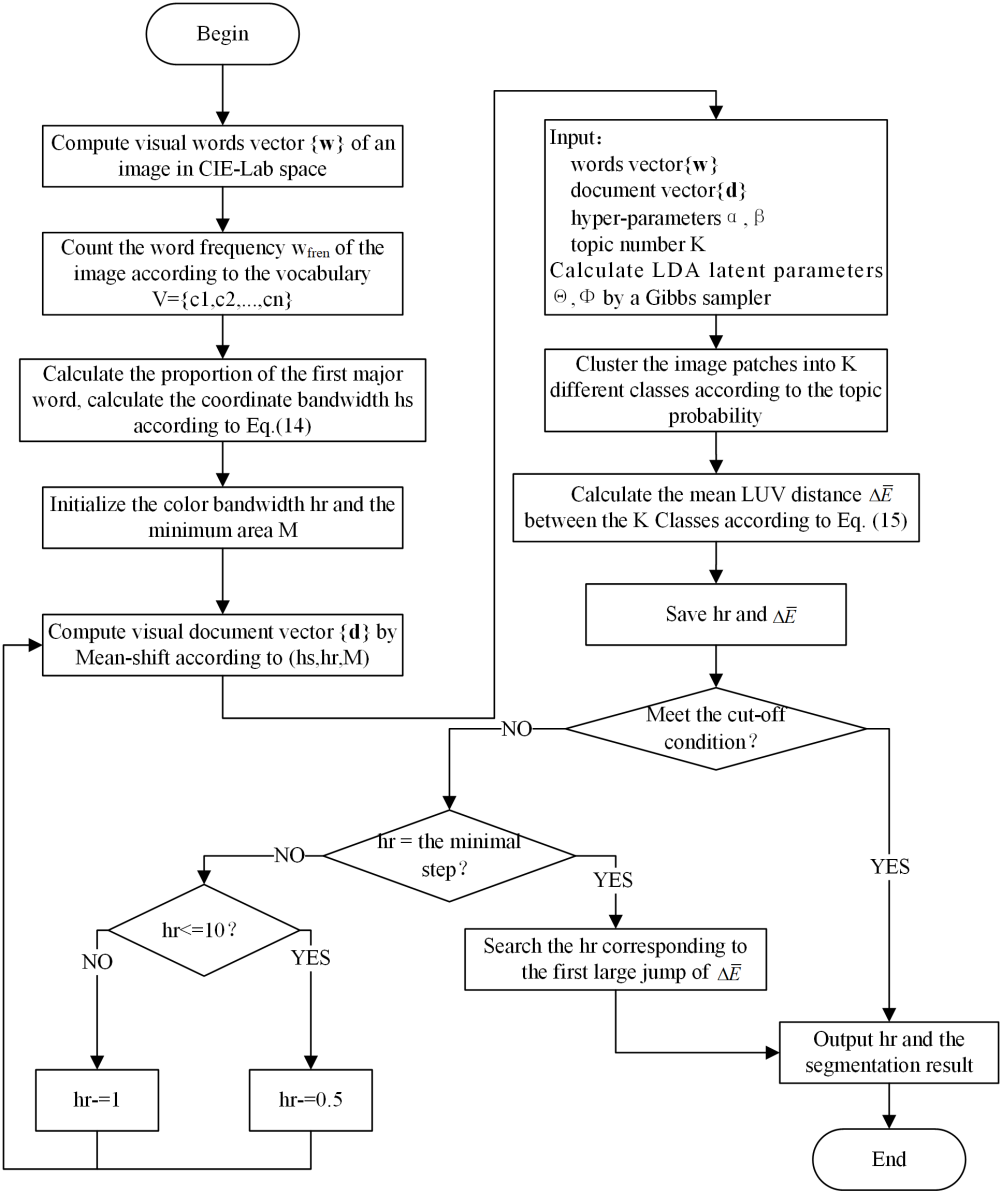
The flowchart of MSBS-LDA segmentation algorithm.

With the help of the Gaussian filter bank, we increase the diversity of words to ensure the local description ability of the LDA algorithm along with a rapid convergence of the unsupervised learning dictionary. The word-document assignment based on Mean-shift improves the image data modelling of documents and the tolerance to different features of the same object, thereby improving the global summary ability of the spatial LDA algorithm. Based on LDA’s word frequency statistics and structural features of segmentation result, Mean-shift is guided to automatically find the optimal bandwidth parameters. The combination of the three processes improves the ability of LDA to solve image segmentation problems. Fig. 2 shows the flowchart of the MSBS-LDA algorithm.

## Plant Segmentation and Leaf Segmentation

The segmentation of greenhouse plant images is divided into two processes: (1) plant extraction from background and (2) single leaf segmentation. The superiority of MSBS-LDA to describe local details in a complex scene and its tolerance to differences in features of the same object make it suitable for the problem of plant segmentation under the greenhouse scene, especially in the case of complex lighting, leaf lesions and mosses. Therefore, we first adopt MSBS-LDA to handle plant segmentation, extracting plants from the complex scene.

**Figure 3:**
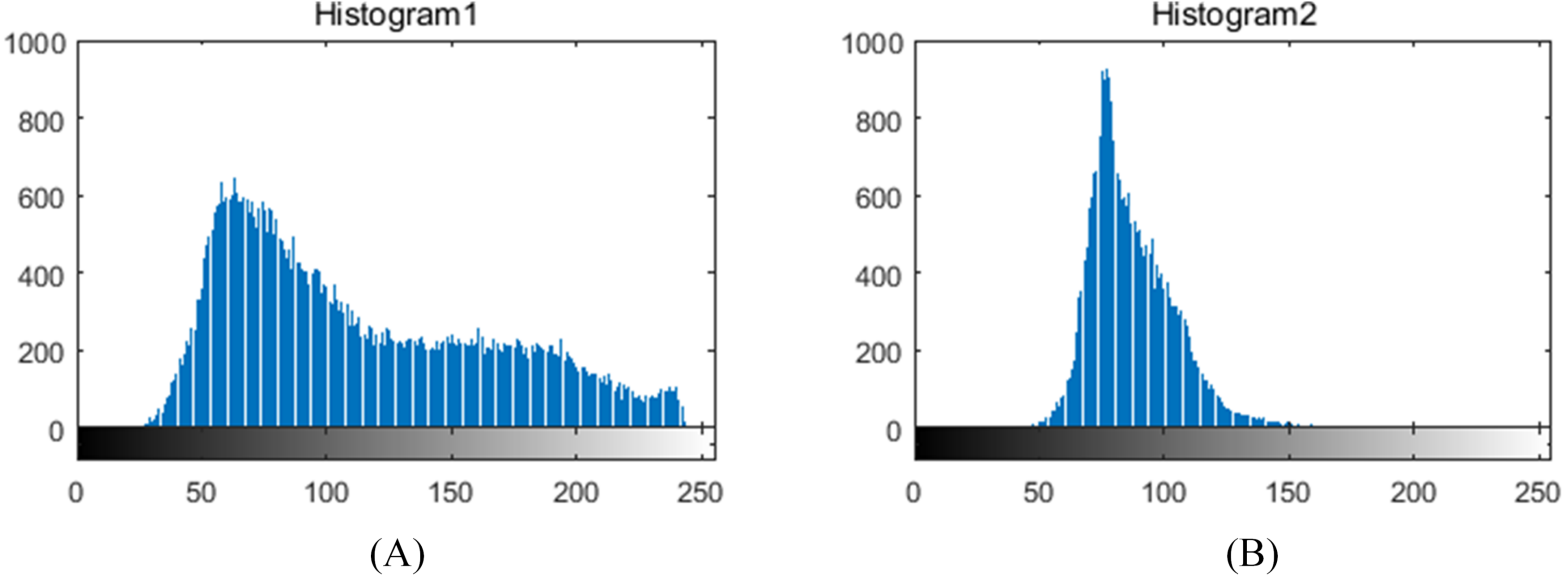
Grayscale histograms of foreground leaf images under different illumination conditions in greenhouses. (A) histogram of uneven illumination image, (B) histogram of even illumination image.

The illumination in greenhouses is ever-changing and the images captured during the fruits bearing period sometimes include tomato fruits of various growth stages, which have a negative impact on leaf segmentation. In these regards, we first compute color difference (R-B) on plant foreground with fruit and then perform threshold segmentation to remove the fruit part. Thereafter, the lighting environment is investigated based on the gray distribution of the leaf foreground histogram, as shown in Fig. 3. The grayscale distribution rate *α*′ is calculated as:

(16) \begin{eqnarray*}{\alpha }^{{^{\prime}}}= \frac{{C}_{hist} \left( {M}_{s}-{R}_{s},{M}_{s}+{R}_{s} \right) }{{C}_{hist} \left( 1,255 \right) } ,{R}_{s}=255\times {\beta }^{{^{\prime}}}/2\end{eqnarray*}

where *C*_*hist*_ is a grayscale statistical function, *M*_*s*_ is the maximum gray value of the accumulated grayscale, R_*s*_ is a grayscale radius, and *β*′ represents a grayscale distribution range. We set *β*′ and the threshold of *α*′ as 0.2 and 0.8, respectively. Equation (16) denotes that if more than 80% of the pixels are distributed within 20% of the grayscale range, the lighting environment of the foreground leaf image is considered to be ideal, otherwise, illumination correction of homomorphic filtering is required to weaken its effect.

**Figure 4:**
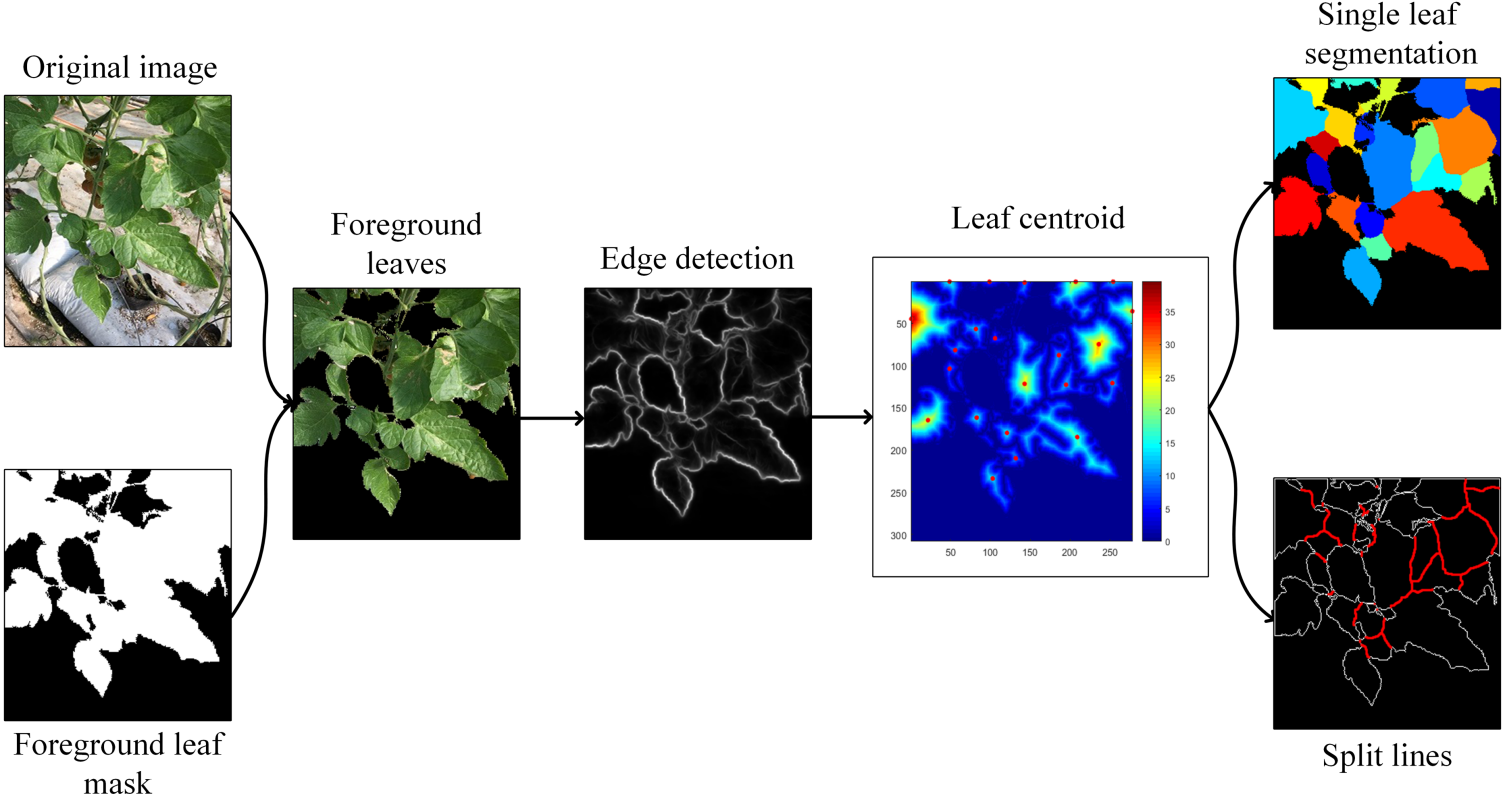
The segmentation process.

**Figure 5:**
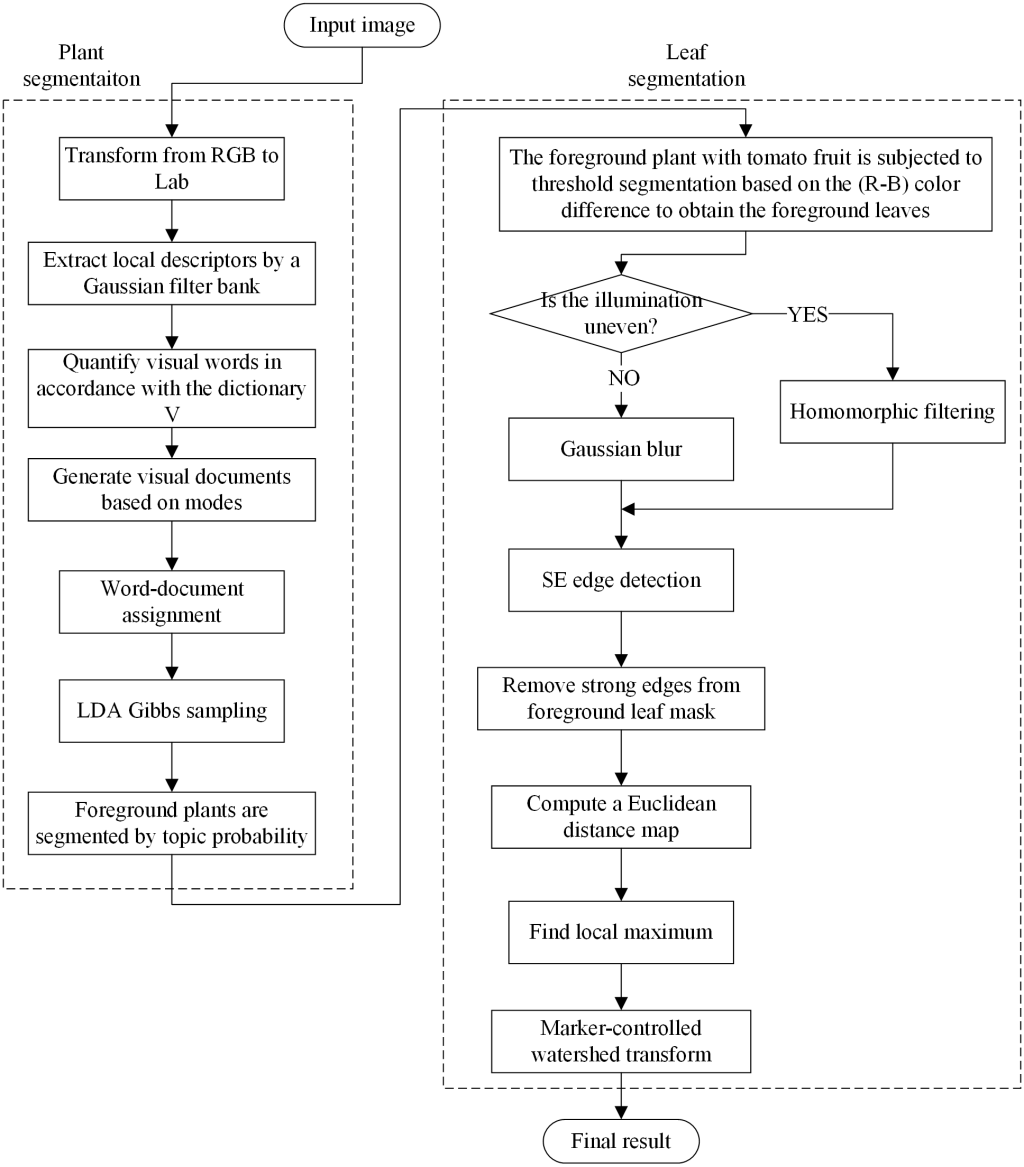
The flowchart of the proposed segmentation method.

Then, leaf segmentation is carried out on the extracted leaf part after illumination response in three steps: strong edge detection, leaf centroid location, and marker-controlled watershed segmentation. Removal of strong edges from foreground leaf mask is a preprocessing step for leaf center detection, which can significantly improve the efficiency and accuracy of local maximum filtering. In the literature, some scholars have provided new ideas for closed contour effects (Valliammal & Geethalakshmi, 2011; Sampath & Shan, 2007; Dollár & Zitnick, 2015; Ming, Li & He, 2012). To decrease algorithm complexity and meet the demand of rapidity, the Structured Edge (SE) detector (Dollár & Zitnick, 2015) is applied to obtain leaf-leaf boundaries. Then we subtract the detected edges from the foreground leaf mask and compute a Euclidean distance map on it. Afterwards, the local maximum of the distance map is searched to locate each leaf’s centroid based on the dilation operation. Further segmentation by watershed algorithm is conducted as the post-process step. The schematic diagram of the segmentation process is shown in Fig. 4 and the overall flowchart of the proposed plant segmentation and leaf segmentation method is shown in Fig. 5.

## Experiments and Analysis

In our research, we first test MSBS-LDA on the images from the Microsoft Research Cambridge Object Recognition Image Database v2 (MSRC-v2) (Microsoft, 2000) to evaluate its performance on the general dataset. For the segmentation of plant and leaf, all the tomato plant images were taken under real greenhouse conditions from three Venlo greenhouses of the Chongming Base of National Facility Agricultural Engineering Technology Research Center (abbreviated as CM), the Sunqiao Modern Agricultural Development Zone in Shanghai (abbreviated as SQ), and the Jiading Experimental Greenhouse in Tongji University (abbreviated as JD). The main characteristic of the CM images is that they have uniform illumination and the plant is in vegetal stage with smooth leaves; while for the SQ images, the plant is in the stage of blossoming and bearing fruits, and the complicated light environment causes reflection as well as shadows on the leaves. The leaves with light or serious disease spots of the JD images increase the diversity of the plant images. It deserves pointing out that all the ground-truths and training set for comparison experiments were labelled manually by the first author. In addition to the tomato plant images, we took 13 images (among which, nine images contain obvious mosses) of Arabidopsis from the Computer Vision Problems in Plant Phenotyping (CVPPP) dataset A1 subset (Scharr et al., 2014; Minervini, Abdelsamea & Tsaftaris, 2014) to test the ability of MSBS-LDA to handle mosses. All experiments were conducted on a PC HP-g4-1059TX machine (Shanghai, China) with 2.10 GHz CPU and 6GB RAM.

### Evaluation metrics

In order to quantitatively evaluate the accuracy of the spatial LDA segmentation algorithms including NR-LDA, OR-LDA, SLIC-LDA and MSBS-LDA, we define the following three metrics:

(1) SA (Segmentation Accuracy) measures the area of overlap between ground-truth and algorithm result:

(17) \begin{eqnarray*}SA= \frac{1}{K} \sum _{i=1}^{K} \frac{ \left\vert {P}_{i}^{gt}\cap {P}_{i}^{ar} \right\vert }{ \left\vert {P}_{i}^{gt} \right\vert } \end{eqnarray*}

(2) OR (Over-segmentation Rate) measures the area of algorithm result not in ground-truth:

(18) \begin{eqnarray*}OR= \frac{1}{K} \sum _{i=1}^{K} \frac{ \left\vert {P}_{i}^{ar} \right\vert - \left\vert {P}_{i}^{gt}\cap {P}_{i}^{ar} \right\vert }{ \left\vert {P}_{i}^{gt} \right\vert } \varepsilon \left( \left\vert {P}_{i}^{ar} \right\vert - \left\vert {P}_{i}^{gt}\cap {P}_{i}^{ar} \right\vert \right) \end{eqnarray*}

(3) UR (Under-segmentation Rate) measures the area of ground-truth not in algorithm result:

(19) \begin{eqnarray*}UR= \frac{1}{K} \sum _{i=1}^{K} \frac{ \left\vert {P}_{i}^{gt} \right\vert - \left\vert {P}_{i}^{gt}\cap {P}_{i}^{ar} \right\vert }{ \left\vert {P}_{i}^{gt} \right\vert } \varepsilon \left( \left\vert {P}_{i}^{gt} \right\vert - \left\vert {P}_{i}^{gt}\cap {P}_{i}^{ar} \right\vert \right) \end{eqnarray*}

where ${P}_{i}^{gt}$ and ${P}_{i}^{ar}$ denote the ground-truth and the algorithm result of the *i*th class, and ε(⋅) is the step function. All of these metrics are counted in pixels, with larger values of SA, smaller values of OR and UR representing higher agreement between ground-truth and algorithmic results.

The metrics above are suitable for the evaluation of general multi-class classifiers, but for plant and leaf segmentation, more targeted quantitative evaluation metrics are required. In this regard, some evaluation criteria proposed in Scharr et al. (2016) are adopted here, which are, respectively, defined as follows:

(1) FBD (Foreground-Background Dice) is Dice score of foreground plant:

(20) \begin{eqnarray*}Dice \left( \text{%} \right) = \frac{2 \left\vert {P}^{gt}\cap {P}^{ar} \right\vert }{ \left\vert {P}^{gt} \right\vert + \left\vert {P}^{ar} \right\vert } \end{eqnarray*}

which measures the degree of overlap between ground-truth *P*^*gt*^ and segmentation result *P*^*ar*^.

(2) SBD (Symmetric Best Dice) is symmetrical mean Dice of all leaves, as computed by:

(21) \begin{eqnarray*}SBD \left( {L}^{ar},{L}^{gt} \right) =\min \left\{ BD \left( {L}^{ar},{L}^{gt} \right) ,BD \left( {L}^{gt},{L}^{ar} \right) \right\} \end{eqnarray*}

where BD (Best Dice) is defined as:

(22) \begin{eqnarray*}BD \left( {L}^{a},{L}^{b} \right) = \frac{1}{M} \sum _{i=1}^{M}\max _{1\leq j\leq N} \frac{2 \left\vert {L}_{i}^{a}\cap {L}_{j}^{b} \right\vert }{ \left\vert {L}_{i}^{a} \right\vert + \left\vert {L}_{j}^{b} \right\vert } \end{eqnarray*}

(3) Dic (Difference in Count) is difference in leaf number between ground-truth and algorithm result:

(23) \begin{eqnarray*}Dic=#{L}^{ar}-#{L}^{gt}\end{eqnarray*}

(4) —Dic— is absolute value of Dic,

(5) MHD (modified Hausdorff distance) measures accuracy of shapes and boundaries by comparing two sets of points, A and B, on the edge of a leaf:

(24) \begin{eqnarray*}MHD \left( A,B \right) =\max \left\{ D \left( A,B \right) ,D \left( B,A \right) \right\} \end{eqnarray*}

with

(25) \begin{eqnarray*}D \left( A,B \right) = \frac{1}{ \left\vert A \right\vert } \sum _{p\in A}\min _{q\in B} \left\| p-q \right\| .\end{eqnarray*}

**Figure 6:**
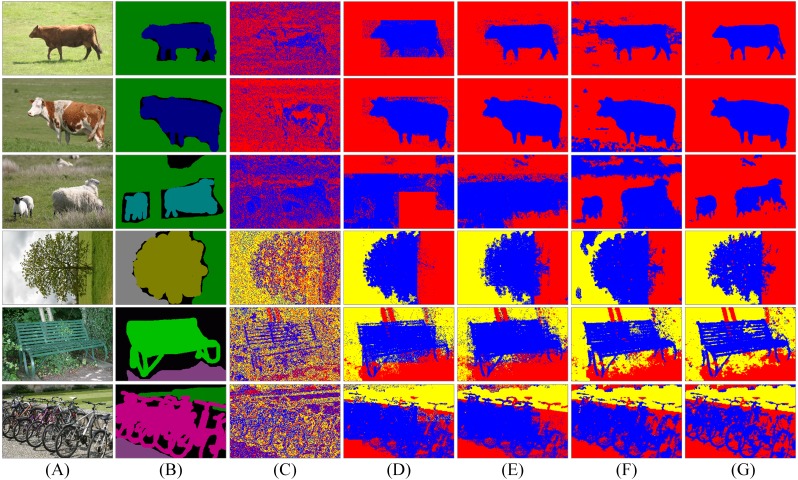
Segmentation results of spatial LDA algorithms with different word-document assignments. Given a collection of images as shown in the column (A), the goal is to segment images into different objects. The column (B) is weakly labelled ground-truth. The columns (C)–(G) are the results of LDA, NR-LDA, OR-LDA, SLIC-LDA and MSBS-LDA, respectively. The original images and their ground-truth credit: the Microsoft Research Cambridge Object Recognition Image Database v2 (MSRC-v2).

**Figure 7:**
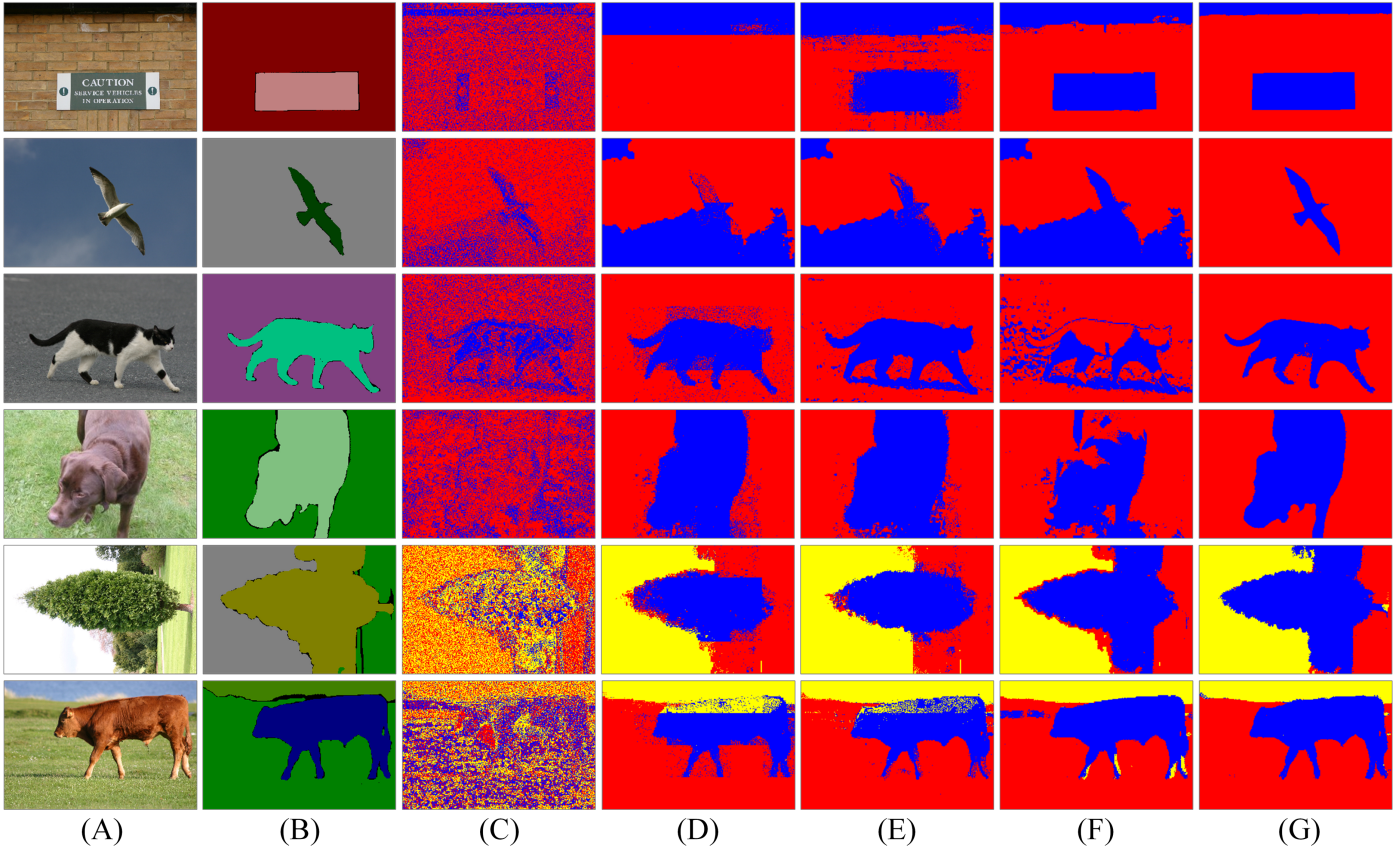
Segmentation results of spatial LDA algorithms on images with high quality ground-truth. The column (A) are original images. The column (B) is high quality ground-truth. The columns (C)–(G) are the results of LDA, NR-LDA, OR-LDA, SLIC-LDA and MSBS-LDA, respectively. The original images and their ground-truth credit: the Microsoft Research Cambridge Object Recognition Image Database v2 (MSRC-v2).

### Spatial LDA evaluation

The experiment is carried out on the MSRC-v2 dataset to test the spatial LDA segmentation algorithms including NR-LDA, OR-LDA, SLIC-LDA and MSBS-LDA.

First, we investigate the influence of different word-document assignment strategies on the performance of spatial LDA. The segmentation results are shown in Figs. 6 and 7. We can figure out that the traditional LDA takes local descriptors as words and the whole image as one document, then it clusters the visual words that often appear in the same image into one object class, which exists two problems: (1) the segmentation result is noisy because spatial information is not considered and (2) since the whole image is treated as one document, if an object is dominant in the image, then other non-dominant objects could be labelled as the dominant one. NR-LDA improves the effect of noise, but documents of non-overlapping regions make rectangle edges noticeable. This kind of borderline situation is further improved by OR-LDA. Compared with spatial structure, SLIC-LDA more meticulously depicts the homogeneity of the atomic regions and the differences between them, so that weakens the tolerance of the documents to the different features in the same object. As a result, some unnecessary edges and noise in the segmentation results are generated. With the description of color and location information of the documents, MSBS-LDA tends to get better segmentation results in both details and global structure. The smooth effect of kernel density estimation and filtering effect of Mean-shift greatly reduce the noise of LDA caused by the BoW model. The results are no longer limited by the size of the rectangular documents, also, the shapes and boundaries are more natural. The evaluation results are shown in Table 1, where MSBS-LDA achieves the highest segmentation accuracy (0.9899), the lowest under-segmentation rate (0.0787) and the lowest over-segmentation rate (0.0101).

**Table 1:** Segmentation evaluation of spatial LDA algorithms for images in Fig. 7.

	SA	OR	UR
LDA	0.7483	0.7072	0.2517
NR-LDA	0.8582	0.7885	0.1419
OR-LDA	0.9509	0.7998	0.0491
SLIC-LDA	0.8976	0.7342	0.1024
MSBS-LDA	** 0.9899**	** 0.0787**	** 0.0101**

**Figure 8:**
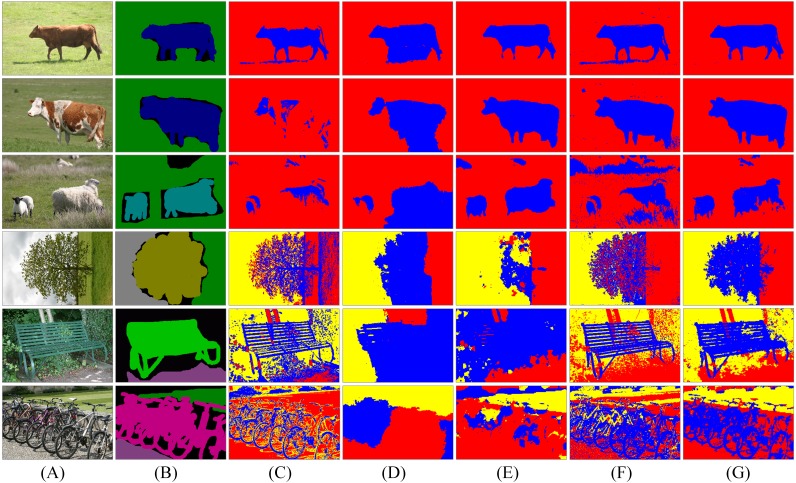
Segmentation results of five unsupervised algorithms. The column (A) are original images. The column (B) is weakly labelled ground-truth. The columns (C)–(G) are the results of 2D-OTSU, Normalized-cut, Co-segmentation, ICM and MSBS-LDA, respectively. The original images and their ground-truth credit: the Microsoft Research Cambridge Object Recognition Image Database v2 (MSRC-v2).

Afterwards, we compare MSBS-LDA with some unsupervised learning segmentation methods. The segmentation results are shown in Fig. 8. It can be seen that MSBS-LDA shows superior performance in general. Compared with 2D-OTSU and ICM, MSBS-LDA can significantly improve the noise situation, and it also has better tolerance to feature differences of the same object. Meanwhile, compared with Normalized-cut and Co-segmentation, the description of the details of MSBS-LDA has not been weakened by the tolerance.

### Plant segmentation evaluation

**Figure 9:**
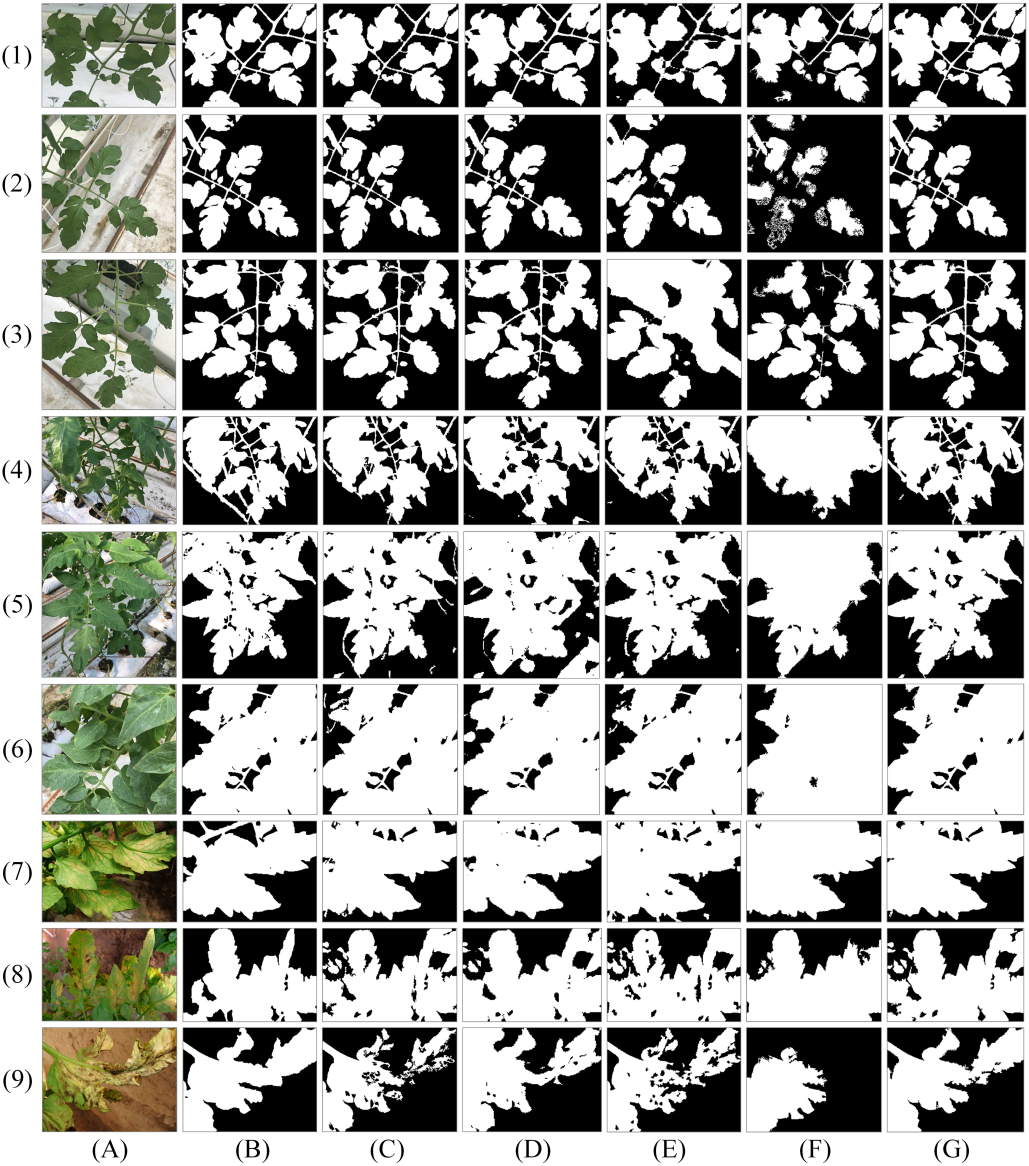
Results of five plant segmentation methods on three tomato plant image sets. The column (A) are original images. The column (B) are ground-truth images labelled by the first author. The columns (C)–(G) are the results of BP, SLIC-OTSU, Co-segmentation, Dense-CRF, and MSBS-LDA, respectively. Original images in the rows (1)–(3) are from Chongming Greenhouse, those in the rows (4)–(6) are from Sunqiao Greenhouse and in the rows (7)–(9) are from Jiading Experimental Greenhouse.

**Figure 10:**
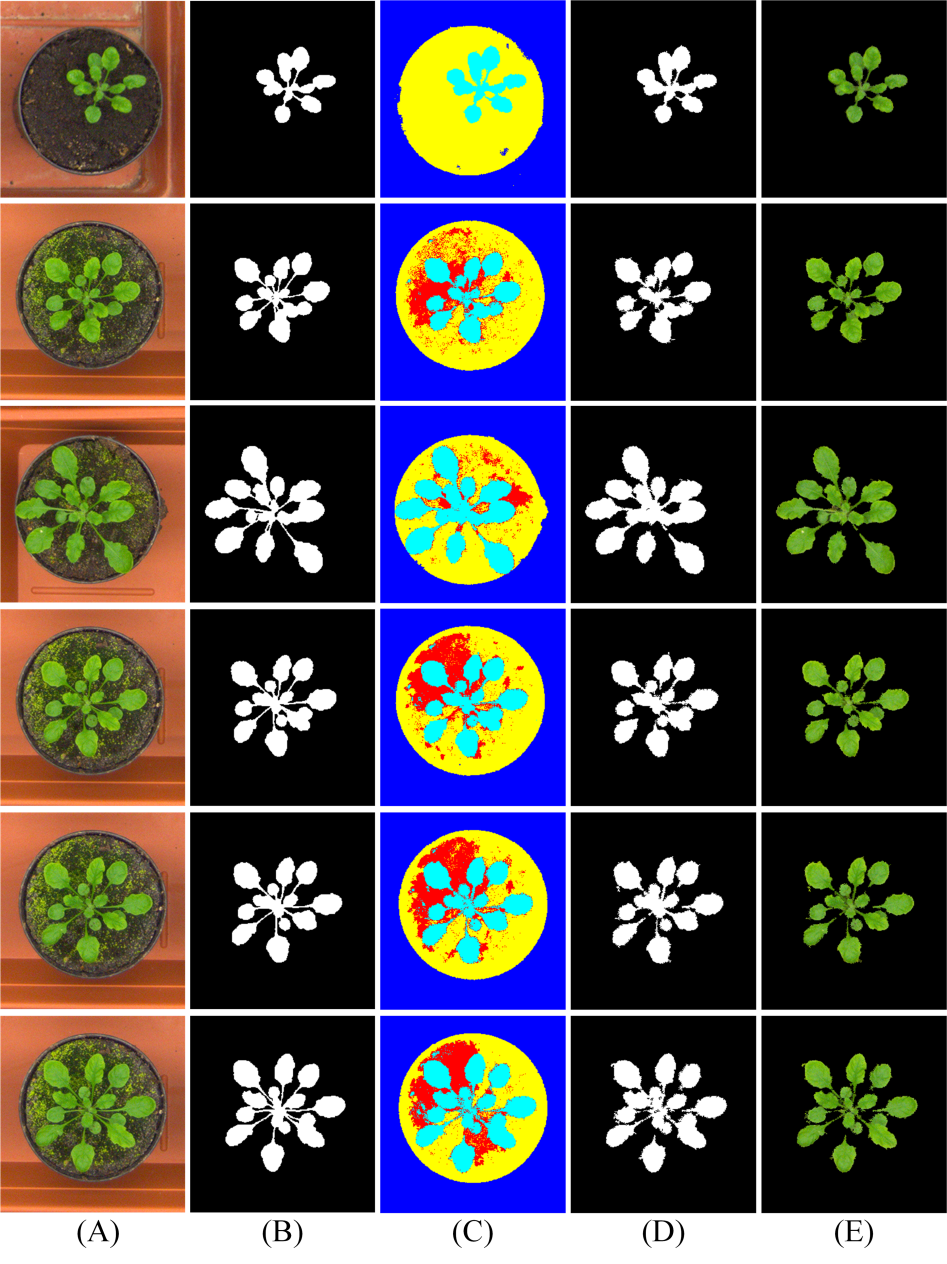
Results of MSBS-LDA on CVPPP A1 subset. The column (A) are original images. The column (B) is high quality ground-truth. The column (C) are the results of MSBS-LDA, the column (D) are the foreground plant masks and the column (E) are the extracted foreground plants. The original images and their ground-truth credit: the CVPPP dataset (A1).

For three kinds of tomato plant images with distinct characteristics, we adopt MSBS-LDA to fulfill the plant segmentation task. Meanwhile, we test some other segmentation algorithms and features in comparison: (1) BP with 3D color and texture features proposed in Minervini, Abdelsamea & Tsaftaris (2014), (2) OTSU with a* channel color of SLIC super-pixels, (3) co-segmentation, (4) dense-CRF based on texton-boost (Krähenbühl & Koltun, 2011; Yang & Li, 2014). The results of the five foreground segmentation methods on three tomato plant image sets are illustrated in Fig. 9, and the quantitative evaluation is shown in Tables 2 and 3. For Arabidopsis images from CVPPP A1 subset, the results of MSBS-LDA are illustrated in Fig. 10, and the quantitative evaluation is shown in Table 4.

According to the experimental results on tomato plant images, for the CM images, MSBS-LDA obtains the segmentation result comparable to BP and SLIC-OTSU, the stems and other details can be well separated from the background. The performance of MSBS-LDA is not affected by the complex background of the SQ image, and there is almost no noise in the result. Moreover, MSBS-LDA shows superior segmentation performance on the JD image which is most difficult for plant extraction. From Fig. 9 it is clear that not only the plant and the background can be effectively separated, but also the leaf disease spots can be accurately included in the foreground plant. When compared with the other four segmentation methods, MSBS-LDA achieves better segmentation accuracy, leaf shape and edge precision in general. In fact, through experiments we also find that gmentation performance of some methods, such as BP and co-segmentation which rely on the training set or the same type of objects in a group, could be greatly affected if the characteristic between images are quite different or the design of the training set is not appropriate. In addition, from Table 5 we can assume that the time consumption of MSBS-LDA is competitive and practicable for most greenhouse applications. From the experimental results on CVPPP A1 subset, we can see that MSBS-LDA is able to separate mosses and plants effectively by adjusting the topic number. The appearance of mosses does not affect the performance of the algorithm much.

**Table 2:** FBD of five methods on three tomato plant image sets.

FBD (for plant, %)	BP	SLIC-OTSU	Co-segmentation	Dense-CRF	MSBS-LDA
CM1	97.90	**98.50**	93.00	92.76	98.08
CM2	**97.83**	97.65	89.44	80.65	97.54
CM3	97.31	97.12	82.04	90.47	**98.02**
SQ1	96.32	96.03	92.44	85.78	**96.42**
SQ2	97.07	96.93	88.99	92.63	**97.44**
SQ3	98.80	99.13	97.15	96.18	**99.18**
JD1	89.17	86.65	89.07	88.85	**90.78**
JD2	94.00	92.02	92.89	92.62	**94.31**
JD3	84.93	90.82	84.31	72.19	**96.54**

**Table 3:** MHD of five methods on three tomato plant image sets.

MHD (for plant)	BP	SLIC-OTSU	Co-segmentation	Dense-CRF	MSBS-LDA
CM1	0.71	**0.48**	5.92	7.04	1.48
CM2	0.46	0.72	1.89	3.14	**0.44**
CM3	**1.51**	2.51	6.90	6.41	2.03
SQ1	4.88	6.88	9.90	8.22	**3.51**
SQ2	6.43	4.17	14.79	5.05	**2.10**
SQ3	3.83	2.23	4.66	2.13	**0.45**
JD1	8.80	9.93	10.36	**8.12**	8.75
JD2	3.92	5.17	3.71	4.36	**3.02**
JD3	7.67	4.69	6.86	32.55	**2.35**

**Table 4:** Scores of MSBS-LDA on CVPPP A1 subset.

Samples	FBD (%)	MHD
13	95.99	0.51
59	95.69	0.62
91	95.70	0.98
96	95.55	0.70
115	95.24	0.70
153	94.54	0.88

**Table 5:** Time complexity of five methods for each image.

Methods	BP	SLIC-OTSU	Co-segmentation	Dense-CRF	MSBS-LDA
Time consumption (s)	22.51	5.51	213.14	166.90	141.64

### Leaf segmentation evaluation

**Figure 11:**
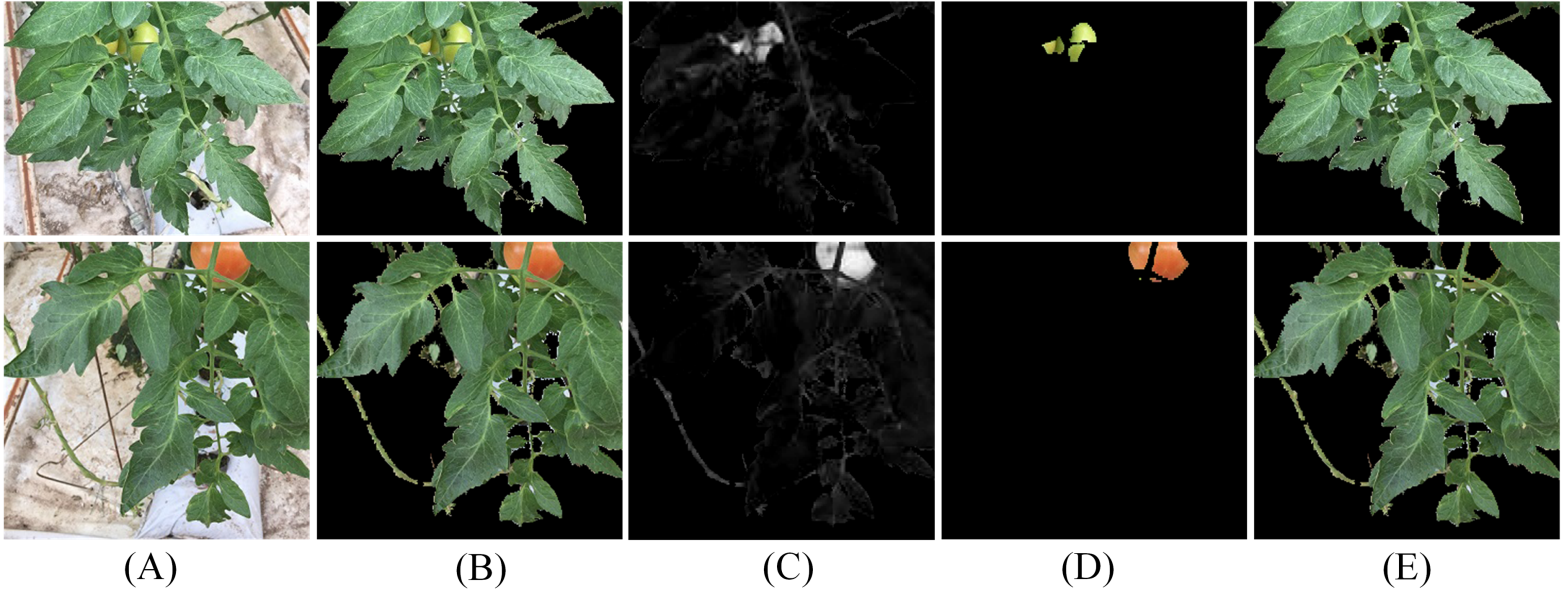
Results of leaf and fruit separation. The column (A) are original images containing fruit, the column (B) are the plant segmentation results, the column (C) are the R-B color differences, the column (D) are the extracted fruit part, and the column (E) are the extracted leaf part.

**Figure 12:**
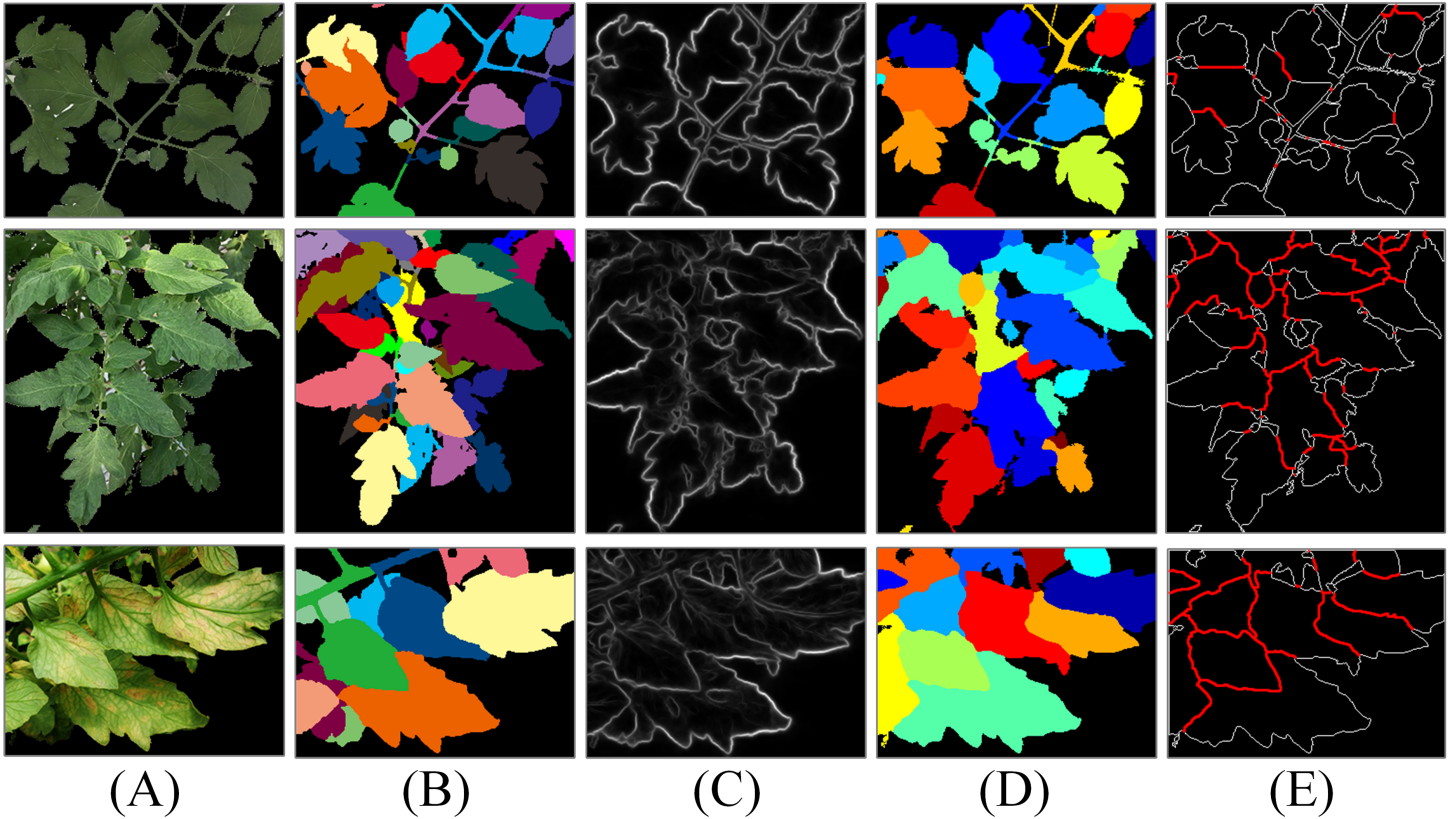
Results of leaf segmentation on three tomato plant image sets. The column (A) are original images, the column (B) are ground-truth images labelled by the first author, the column (C) are the SE detector results, the column (D) are the leaf segmentation results and the column (E) are the split lines.

**Figure 13:**
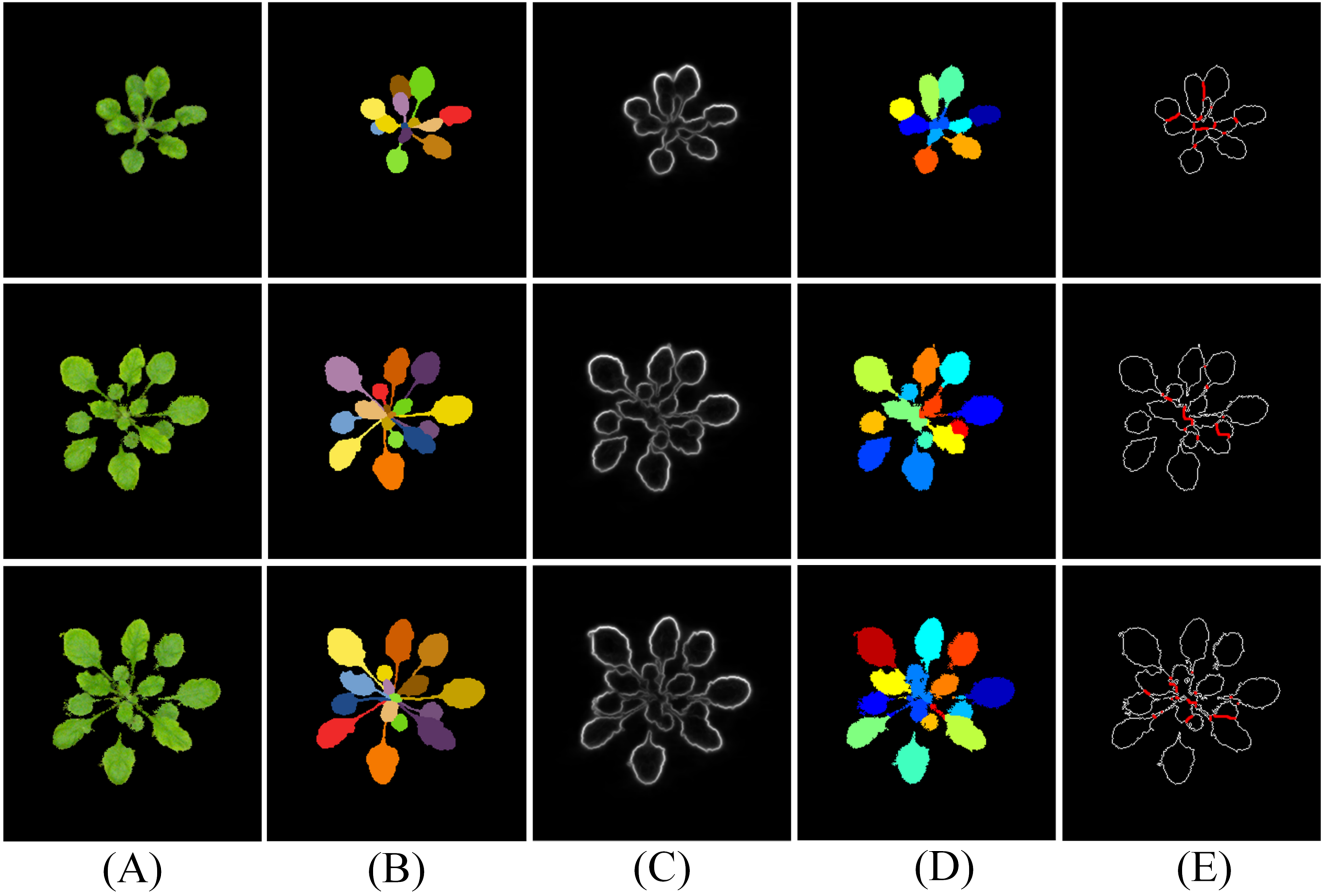
Results of leaf segmentation on CVPPP A1 subset. The column (A) are original images, the column (B) is high quality ground-truth, the column (C) are the SE detector results, the column (D) are the leaf segmentation results and the column (E) are the split lines. The original images and their ground-truth credit: the CVPPP dataset (A1).

For the plant segmentation results containing fruit part, the separation results of leaves and fruits are shown in Fig. 11. The leaf segmentation results on three tomato plant image sets and the CVPPP A1 subset are shown in Figs. 12 and 13, respectively. The evaluation scores for all the testing images are listed in Table 6. For more complicated cases, like the JD images with lesions and the SQ images with both complex lighting environment and seriously overlapped leaves, our method reaches segmentation accuracy ranging from 50% to 60%. For the CM images with more uniform illumination and smooth leaves, the accuracy becomes more than 65%. The leaf segmentation scores of CVPPP A1 images are listed in Table 7. For Arabidopsis images with mosses and weak boundaries between overlapping leaves, the segmentation accuracy is up to 70%.

In this study, we find that if centroids of leaves are not detected correctly owing to serious overlapping, complicated lighting environment or side-effect of lesions, the performance of watershed algorithm will be limited. Besides, low contrast, weak boundaries and complex posture can lead to low segmentation accuracy. The method can also be fine-tuned to achieve a higher accuracy on one particular image, such as utilizing a more accurate edge detector, a more appropriate illumination normalization module or a better local maximum filter.

**Table 6:** Leaf segmentation evaluation on three tomato plant image sets.

Image sets	FBD (for leaves, %)	SBD (%)	|DiC |	DiC
CM	96.77 (1.83)	65.37 (6.25)	1.80 (1.32)	−1.00 (2.05)
SQ	96.75 (1.71)	55.86 (4.00)	2.60 (2.17)	−2.60 (2.17)
JD	93.73 (2.79)	51.88 (7.16)	1.25 (0.89)	1.25 (0.89)
All	95.89 (2.46)	58.12 (8.03)	1.93 (1.63)	−0.93 (2.37)

**Notes.**

Average values are shown for metrics described in ‘Evaluation metrics’ and in parenthesis standard deviation.

**Table 7:** Leaf segmentation evaluation on CVPPP A1 subset.

	FBD (%)	SBD (%)	—DiC—	DiC
Mean	94.42 (1.48)	73.81 (5.32)	2.62 (1.33)	−2.62 (1.33)
Median	95.24	74.71	3.00	−3.00
Max	95.99	82.40	5.00	−1.00
Min	92.03	66.12	1.00	−5.00

## Discussion and Conclusion

In this study, we propose a modified statistical model of LDA, namely MSBS-LDA, to segment greenhouse tomato plants in an unsupervised way, and leaf segmentation is carried out subsequently. Through our experiments in different cases, some conclusions are drawn as follows:

(1) After analyzing the difficulties of using natural language processing model in image segmentation, it is proposed to improve the LDA algorithm in the spatial structure encoding of images through the word-document assignment. The diversity of visual vocabulary is guaranteed by constructing visual words, which can improve the ability of LDA to describe image details. Moreover, the single document mapping is changed by designing visual documents, ameliorating the spatial summarization ability of LDA. The comparison experiments show that the spatial LDA algorithms with word-document assignments outperform the traditional one in image segmentation.

(2) In regards to leaf lesion spots and complicated backgrounds of greenhouses, a spatial LDA segmentation algorithm based on bandwidths searching of Mean-shift documents (MSBS-LDA) is proposed and applied to plant segmentation. The non-parametric estimation is adopted to space modelling, and the modes of Mean-shift are mapped as documents. The comparison experiments show that the proposed MSBS-LDA algorithm can simultaneously give good expression to image details and the global structure information, so that it can accurately include the lesion part in the plant and separate the plant from mosses of similar color through the adjustment of the topic number.

(3) In regards to tomato fruits and ever-changing illumination in greenhouses, an improved watershed segmentation method is proposed and applied to leaf segmentation. The fruit separation, illumination normalization, strong edge detection, leaf centroid location, and marker-controlled watershed segmentation are carried out sequentially to complete leaf segmentation. The experiments show that the method can guarantee a certain segmentation accuracy for three greenhouse tomato plant image sets of different characteristics.

##  Supplemental Information

10.7717/peerj.5036/supp-1 Data S1 Raw data: The greenhouse plant images and their ground-truth, the segmentation results and the code used

10.7717/peerj.5036/supp-2 Data S2 Raw data: The CVPPP images used, their ground-truth and the segmentation results

## Additional Information and Declarations

### Data Availability

The following information was supplied regarding data availability: The raw data are provided in the Supplemental Files.
